# Protocol of the RACB study: a multicenter, single-arm, prospective study to evaluate the efficacy of resection of initially unresectable hepatocellular carcinoma with atezolizumab combined with bevacizumab

**DOI:** 10.1186/s12885-023-11302-6

**Published:** 2023-08-21

**Authors:** Masayuki Okuno, Takamichi Ishii, Akihiko Ichida, Akihiko Soyama, Nobuyuki Takemura, Seiko Hirono, Susumu Eguchi, Kiyoshi Hasegawa, Yasuharu Sasaki, Kohei Uemura, Norihiro Kokudo, Etsuro Hatano

**Affiliations:** 1 Department of Gastroenterological Surgery, Hyogo Medical University, Nishinomiya Hyogo, Japan; 2 Department of Surgery, Graduate School of Medicine, Kyoto University, 54 Kawahara-cho, Shogoin, Sakyo-ku, Kyoto, 606-8507 Japan; 3 Hepato-Biliary-Pancreatic Surgery Division, Department of Surgery, Graduate School of Medicine, The University of Tokyo, Tokyo, Japan; 4 Department of Surgery, Nagasaki University Graduate School of Biomedical Sciences, Nagasaki, Japan; 5 Hepato-Biliary-Pancreatic Surgery Division, Department of Surgery, National Center for Global Health and Medicine, Tokyo, Japan; 6 Department of Data Science, Center for Clinical Sciences, National Center for Global Health and Medicine, JCRAC Data Center, Tokyo, Japan; 7 Department of Biostatistics and Bioinformatics, Interfaculty Initiative in Information Studies, The University of Tokyo, Tokyo, Japan

**Keywords:** Hepatocellular carcinoma, Unresectable, Advanced stage, Conversion, Immune checkpoint inhibitor

## Abstract

**Background:**

Although the standard therapy for advanced-stage hepatocellular carcinoma (HCC) is systemic chemotherapy, the combination of atezolizumab and bevacizumab (atezo + bev) with a high objective response rate may lead to conversion to resection in patients with initially unresectable HCC. This study aims to evaluate the efficacy of atezo + bev in achieving conversion surgery and prolonged progression-free survival (PFS) for initially unresectable HCC.

**Methods:**

The RACB study is a prospective, single-arm, multicenter, phase II trial evaluating the efficacy of combination therapy with atezo + bev for conversion surgery in patients with technically and/or oncologically unresectable HCC. The main eligibility criteria are as follows: (1) unresectable HCC without a history of systemic chemotherapy, (2) at least one target lesion based on RECIST ver. 1.1, and (3) a Child‒Pugh score of 5–6. The definition of unresectable tumors in this study includes macroscopic vascular invasion and/or extrahepatic metastasis and massive distribution of intrahepatic tumors. Patients will be treated with atezolizumab (1200 mg/body weight) and bevacizumab (15 mg/kg) every 3 weeks. If the patient is considered resectable on radiological assessment 12 weeks after initial chemotherapy, the patient will be treated with atezolizumab monotherapy 3 weeks after combination chemotherapy followed by surgery 3 weeks after atezolizumab monotherapy. If the patient is considered unresectable, the patient will continue with atezo + bev and undergo a radiological assessment every 9 weeks until resectable or until disease progression. The primary endpoint is PFS, and the secondary endpoints are the overall response rate, overall survival, resection rate, curative resection rate, on-protocol resection rate, and ICG retention rate at 15 min after atezo + bev therapy. The assessments of safety and quality of life during the treatment course will also be evaluated. The number of patients has been set at 50 based on the threshold and the expected PFS rate at 6 months after enrollment of 40% and 60%, respectively, with a one-sided alpha error of 0.05 and power of 0.80. The enrollment and follow-up periods will be 2 and 1.5 years, respectively.

**Discussion:**

This study will elucidate the efficacy of conversion surgery with atezo + bev for initially unresectable HCC. In addition, the conversion rate, safety and quality of life during the treatment course will also be demonstrated.

**Trial registration:**

This study is registered in the Japan Registry of Clinical Trials (jRCTs051210148, January 7, 2022).

## Background

Hepatocellular carcinoma (HCC) is the sixth most common cancer worldwide and the fourth leading cause of cancer-related death [1]. Potential curative therapies such as resection, liver transplantation, and local ablation for early-stage HCC and transarterial chemoembolization (TACE) for intermediate-stage HCC have been adopted as the standard of care according to several clinical practice guidelines [2, 3]. However, despite recent advances in surveillance and early detection of patients at high risk for HCC occurrence, many patients with HCC present with advanced or metastatic disease at diagnosis or disease recurrence after local interventions with curative intent and are indicated for systemic chemotherapy [4]. Sorafenib is an oral multikinase inhibitor that was the first systemic chemotherapy proven to have survival efficacy as a first-line treatment, with 10.7 months of overall survival (OS) [5]. Lenvatinib has been shown to be noninferior to sorafenib in terms of OS[6]. In 2020, the IMbrave150 study demonstrated the superiority of combination therapy of atezolizumab and bevacizumab (atezo + bev) in survival compared to sorafenib, and atezo + bev has been recommended as a first choice of first-line systemic therapy in patients with advanced HCC [2, 7]. However, 19.2 months of median OS after atezo + bev in the Imbrave150 study is unsatisfactory, and additional treatment strategies, such as conversion surgery for unresectable HCC, are urgently needed for a cure.

Conversion therapy is the treatment strategy used to achieve tumor downstaging and to provide patients with initially unresectable or borderline resectable tumors the opportunity to undergo curative local treatment. Conversion therapy has been widely adopted for various solid tumors, such as metastatic colorectal cancer and pancreatic cancer. As the response rate to chemotherapy correlates with the conversion rate, chemotherapy regimens with a high response rate are recommended for patients with initially unresectable colorectal liver metastases [8]. To date, the feasibility and efficacy of conversion surgery for unresectable HCC remain under debate. As the objective response rate (ORR) of sorafenib treatment was low at 2% in the SHARP study [5], the conversion to resection rate after sorafenib treatment has been reported to be as low as 1.4-5% [9, 10]. In association with an approximately 3 times higher ORR with lenvatinib (18.8%) compared to sorafenib [6], a relatively high conversion rate of 11–60% after lenvatinib therapy has been reported [11, 12]. However, the survival benefit of conversion surgery after lenvatinib has not yet been shown in studies with large case series. Although the high ORR of atezo + bev (29.8%) is expected to contribute to a high conversion rate and lead to prolonged OS, no prospective study focusing on conversion therapy after atezo + bev for initially unresectable HCC has been reported.

## Methods/Design

### Aim

This study (the RACB study) aims to investigate the feasibility and survival efficacy of atezo + bev including subsequent surgical intervention if amenable in patients with initially unresectable HCC. The primary endpoint is progression-free survival (PFS) evaluated with Response Evaluation Criteria in Solid Tumors (RECIST) ver. 1.1. The secondary endpoints are ORR according to RECIST ver1.1 and mRECIST, PFS according to mRECIST, OS, overall resection rate, curative resection rate, on-protocol resection rate, and indocyanine green retention rate at 15 min (ICG-R15) after atezo + bev therapy. Safety and quality of life (QOL) will also be assessed.

### Study design

The RACB study is an open-label, prospective, single-arm, multicenter phase II trial that will be conducted at 16 centers in Japan. The study was registered in the Japan Registry of Clinical Trials (jRCTs051210148, January 7, 2022).

### Inclusion criteria

The inclusion criteria of the current study are as follows.

1. Patients diagnosed with HCC with no prior systemic therapy (sorafenib, lenvatinib, or immunotherapy) for HCC.
2. The presence of at least one target lesion based on RECIST ver1.1.
3. Patients 20 years of age or older.
4. An Eastern Cooperative Oncology Group Performance Status (PS) of 0–1.
5. Adequate organ function*.

*white blood cell ≥ 3000/mm^3^, neutrophil count ≥ 1500/mm^3^, hemoglobin ≥ 8.5 g/dL, platelet count ≥ 60,000/mm^3^, total bilirubin ≤ 3.0 mg/dL, serum albumin ≥ 2.8 g/dL, aspartate aminotransferase and alanine aminotransferase ≤ 5 times the upper limit of each medical institution (excluding cases associated with cancer), serum creatinine ≤ 1.5 times the upper limit of each medical institution, urine protein ≤ 2+, and urine protein/creatinine ratio < 2.0 at any time when urine protein is ≥ 3+ (urine protein < 2.0 g in case of 24-hour urine collection).

6 A Child‒Pugh score of 5–6 within 14 days prior to enrollment.
7 No other active malignancies.
8 The patient’s written informed consent will be obtained after the patient receives a sufficient explanation of the study.
9 Unresectable HCC meeting the following criteria: A, B, C, D, or E.

A) [Intrahepatic vascular invasion] Intrahepatic tumor with vascular invasion to either the right middle and left hepatic vein main trunks, the inferior right hepatic vein, the short hepatic vein or the inferior vena cava, portal invasion in the secondary branch of the portal vein or more, or biliary invasion in the secondary branch of the bile duct or more. No extrahepatic tumor.
B) [Synchronous extrahepatic metastases] Resectable intrahepatic tumors without gross vascular invasion, and the presence of extrahepatic tumors such as distant organ metastases, peritoneal metastases, lymph node metastases, and tumor dissemination along the puncture route*.
C) [Intrahepatic vascular invasion and synchronous extrahepatic metastasis] Intrahepatic tumors with vascular invasion and synchronous extrahepatic metastasis*.
D) [Macroscopic residual tumor] When complete resection of the intrahepatic tumor is difficult but therapeutically meaningful surgical resection is performed, expecting a survival benefit or improvement in the QOL.
E) [Metachronous extrahepatic metastases] Metachronous extrahepatic metastases* with no or controllable intrahepatic tumor.

*Limited to single organ metastasis. Adrenal gland: no bilateral metastasis. Lymph nodes: limited to intra-abdominal lymph nodes. The upper limit of the number of tumors is 5 in lung metastases, 2 in lymph node metastases, 5 in peritoneal metastases, 1 in bone metastasis, and 1 in paracentesis route recurrence. When no definite diagnosis of extrahepatic metastasis is obtained, the eligibility of patients for the study should be determined comprehensively at each institution following appropriate examinations, such as positron emission tomography.

### Exclusion criteria

The exclusion criteria of the current study are as follows.

1. Patients receiving full doses of oral or parenteral anticoagulants or thrombolytics intended for treatment (not for prophylaxis) and those who receive them within 10 days prior to enrollment.
2. Having untreated or not adequately treated esophageal varices and/or varices with or at high risk of bleeding, or a having history of bleeding due to esophageal varices and/or varices within 180 days prior to enrollment.
3. A history of thrombosis and/or embolism within 180 days prior to enrollment.
4. Patients undergoing major surgical procedures, open wound biopsy or suture procedures for major trauma within 28 days prior to enrollment and those who will receive major surgical procedures during the study period.
5. Patients receiving systemic immunostimulatory agents (e.g., interferon, interleukin-2) within 180 days prior to enrollment.
6. Patients receiving systemic immunosuppressants within 14 days prior to enrollment and those expected to receive systemic immunosuppressants during the study period, except for mineralocorticoids or corticosteroids as the treatment for chronic obstructive pulmonary disease or asthma.
7. A history and complications of autoimmune disease within 180 days prior to enrollment.
8. Synchronous double cancer or metachronous double cancer with a disease-free interval < 3 years, excluding early-stage cancers with a low risk of recurrence, such as cervical carcinoma in situ, basal cell carcinoma, superficial bladder tumor, early esophageal cancer, early gastric cancer, and early colorectal cancer.
9. Participation in another study of unapproved drugs or a study requiring intervention (including a follow-up period).
10. Critical cardiovascular disease (New York Heart Association Class II or higher heart disease, myocardial infarction), unstable arrhythmia or unstable angina in the 90 days prior to enrollment.
11. Pregnant, breastfeeding, positive pregnancy test (pregnancy tests conducted in menstruating women within the past year), and women and men unwilling to use contraceptives during the study period.
12. Clinically uncontrolled pleural effusion, pericardial effusion or ascites.
13. Complications of hepatic encephalopathy.
14. Serious complications as listed below: uncontrolled hypertension with or without antihypertensive medication; severe infections occurring within 28 days before enrollment, excluding hepatitis B virus and C virus; receiving hemodialysis due to renal failure; having a serious mental illness; or having an allergic reaction to contrast medium interfering with angiography.
15. A history of serious allergic or anaphylactic reactions to chimeric antibody, humanized antibody, or fusion protein.
16. A history of hypersensitivity to either Chinese hamster ovary cell-derived products or components of atezolizumab or bevacizumab products.
17. A history of hemorrhagic disease, gastrointestinal hemorrhage or active hemoptysis.
18. Difficulty ingesting medication.
19. Human immunodeficiency virus-positive.
20. Active pulmonary fibrosis or interstitial pneumonia.
21. A history of blood transfusions or receiving granulocyte colony stimulating factor products within 14 days prior to enrollment.
22. Poor general condition and the attending physician judges that the patient is not suitable for participation in the study.

### Protocol treatment

**Fig. 1:**
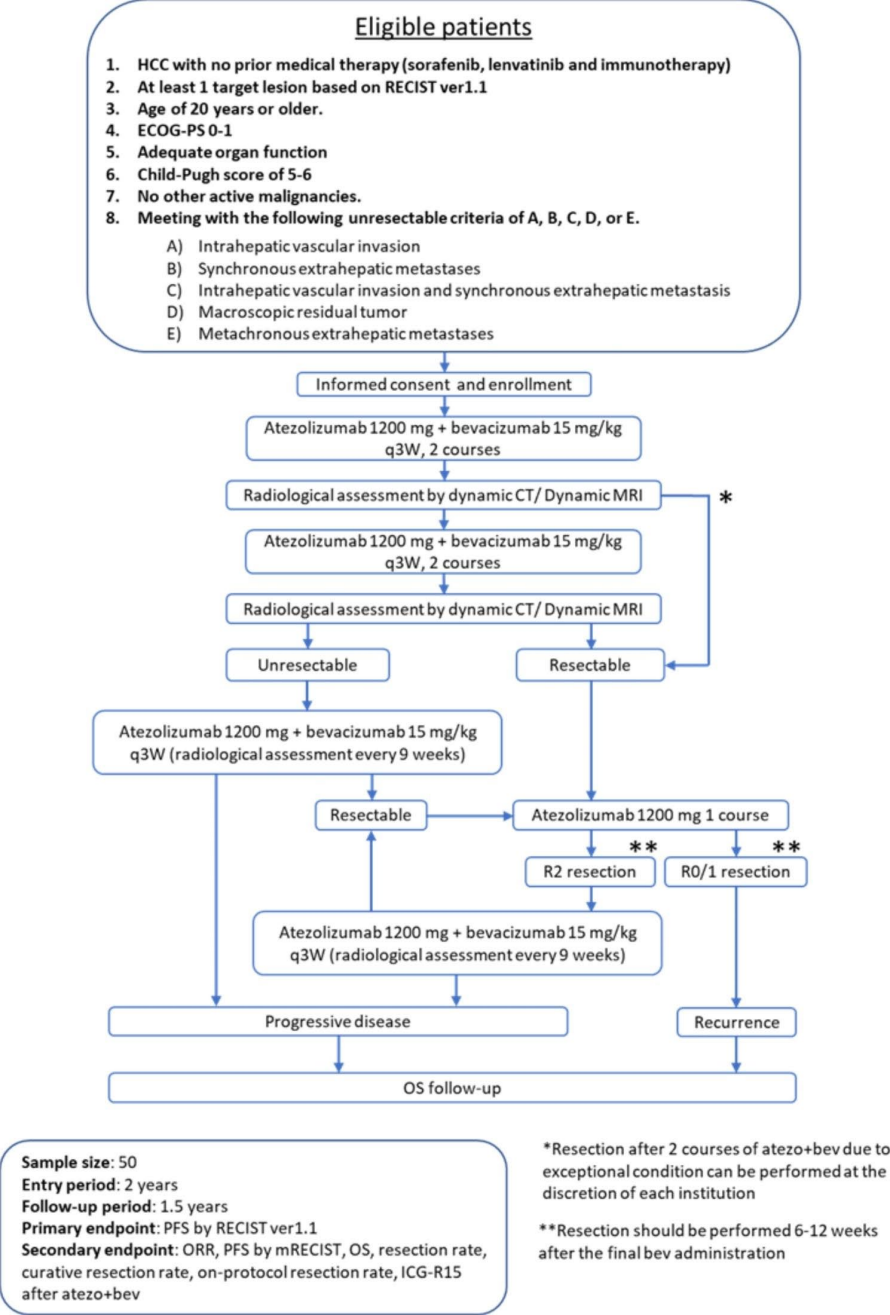
Study schema of the RACB study HCC: hepatocellular carcinoma; RECIST: Response Evaluation Criteria in Solid Tumors; ECOG–PS: Eastern Cooperative Oncology Group–performance status; CT: computed tomography; MRI: magnetic resonance imaging; OS: overall survival; PFS: progression-free survival; ORR: objective response rate; ICG-R15: indocyanine green retention rate at 15 min

Figure 1 shows the schema of the RACB study. Patients will be treated with atezolizumab (1200 mg/body weight) and bevacizumab (15 mg/kg) intravenously every 3 weeks on Day 1. Radiological assessment on contrast-enhanced dynamic computed tomography (CT) or magnetic resonance imaging (MRI) will be performed 6 and 12 weeks after the initial administration of atezo + bev, and conversion surgery will be performed 6–12 weeks after the final administration of atezo + bev if the tumors become resectable. Conversion surgery will be performed after 4 courses of atezo + bev in principle but can be performed after 2 courses at the discretion of the researchers if the tumors are converted to resectable tumors and there are reasons for the termination of atezo + bev, such as difficulty with maintaining atezo + bev therapy due to side effects. Once the tumors are considered to be resectable, atezolizumab single therapy will be performed 3 weeks after the final administration of atezo + bev. In cases of unresectable tumors at radiological assessment, atezo + bev treatment will be continued until resection or until progression of the disease (PD). Adjuvant chemotherapy will not be given to patients whose tumors are curatively resected. When the tumors are not completely resected, atezo + bev will be administered until they become resectable or until PD.

### Assessment

Radiological assessment on contrast-enhanced dynamic CT or MRI will be performed every 9 weeks after the second radiological assessment (at 12 weeks) until PD, regardless of conversion surgery. The patients will be followed-up for 18 months after study inclusion. PFS is defined as the time from enrollment to PD or death according to RECIST version 1.1 and mRECIST. The ORR is defined as the percentage of patients relative to the total number of enrolled subjects who achieve a complete response (CR) or partial response (PR) based on imaging according to RECIST version 1.1 and mRECIST. OS is defined as the time from enrollment to death. The surgical resection rate is defined as the percentage of patients relative to the total number of enrolled subjects who undergo conversion surgery regardless of curability. The curative resection rate is defined as the rate of patients who undergo curative conversion surgery (R0 or R1 resection) among all patients. The on-protocol resection rate is defined as the percentage of patients who undergo conversion surgery based on the radiological assessment of CR, PR or SD. ICG-R15 will be assessed before and 12 weeks after the initial administration of atezo + bev and then before surgery if surgical resection will be performed at a time other than 12 weeks after initial atezo + bev. The safety of the current protocol treatment will be evaluated based on the Common Terminology Criteria for Adverse Events (CTCAE) version 5.0. A QOL assessment will be performed according to the European Organization for Research and Treatment of Cancer Quality of Life Questionnaire Core 30 (EORTC-QLQ-C30), the EORTC-QLQ-HCC18, and the EuroQoL 5 dimension 5 level (EQ-5D-5 L). Postoperative complications will be assessed based on the Clavien‒Dindo classification [13].

### Study design and statistical considerations

The 6-month PFS according to RECIST version 1.1 in the IMbrave150 study was 54.5% [7]. Because the patients eligible for the current study have worse prognostic factors than those in the IMbrave150 study, the null PFS probability at six months is 40%, and the alternative PFS probability at 6 months is 60%. The hypothesis was assessed using the exact binomial test of a one-sided alpha level of 0.05, and a power of 0.80 was calculated. Therefore, the sample size was calculated as 47. Considering dropouts, a total of 50 patients will be enrolled. The enrollment period will be 2 years, and the follow-up period will be 1.5 years from the final enrollment.

### Safety and efficacy assessment

An independent committee will review data on the safety and efficacy of the initial 6 cases and inform a primary safety report to the principal investigator. Accordingly, the principal investigator will determine the modification of the study protocol or termination of the study based on the report of the committee.

## Discussion

The Barcelona Clinic Liver Cancer (BCLC) strategy for prognosis prediction and treatment recommendation classifies HCC patients into groups from very early stage (0) to terminal stage (D) disease based on tumor burden, the extent of the underlying liver disease, and PS. The intermediate stage B group includes patients with multinodular, preserved liver function and a PS of 0. A subgroup of BCLC-B, including patients with diffuse, infiltrative, extensive HCC liver involvement, and the advanced stage C group, including patients presenting with vascular invasion or extrahepatic spread, PS ≤ 2, and preserved liver function, are recommended to undergo systemic therapy [2]. Although the current systemic chemotherapy regimens being used as first-line therapies, such as atezo + bev and tremelimumab plus durvalumab, have shown superiority in OS compared to sorafenib, these systemic therapies cannot lead to a cure in patients who are not amenable or not suitable for locoregional therapy [7, 14]. As the goal for cancer treatment is to remove the tumor and minimize recurrence, leading to cancer-free or chemotherapy-free, advances in treatment, such as conversion therapy for advanced HCC, are in high demand.

Conversion therapy may be an option for the treatment of advanced HCC and has the potential for cure. However, little is known about the feasibility and efficacy of this strategy for HCC. Although several studies regarding conversion surgery after sorafenib treatment have been reported, the conversion to resection rate of these studies is only 1.4-5% [9, 10]. As lenvatinib shows a higher response rate than sorafenib with similar survival efficacy, conversion surgery after lenvatinib therapy is expected. A high conversion rate of 11–60% after lenvatinib therapy has been reported [11, 12], but the survival efficacy of conversion surgery after lenvatinib is uncertain. Another treatment option before conversion surgery is combination immunotherapy. Because the survival efficacy of atezo + bev for advanced HCC was shown in 2020, the feasibility and efficacy of conversion surgery after atezo + bev have not been reported. A prior retrospective study regarding combination immunotherapy followed by surgery described a 16% conversion rate, and also showed that all patients whose tumor was evaluated as a partial response on radiological assessment had a complete pathological response [15]. Therefore, conversion therapy after atezo + bev also raises high expectations for a cure or prolonged survival.

One of the particular concerns during conversion therapy with atezo + bev is safety, especially in cases requiring major hepatectomy. A prior study on conversion therapy after combination immunotherapy showed a case of mortality due to postoperative liver failure [15]. In the current study, patients will continuously undergo liver function tests during atezo + bev. ICG-R15 will also be performed before atezo + bev, after 4 courses of atezo + bev, and preoperatively to ensure the safety of conversion surgery. In addition, the first 6 cases will be included from the 5 institutions that have sufficient experience with conversion surgery for advanced HCC after multikinase inhibitors, and these 6 cases will be reviewed by an independent safety and efficacy committee. After the submission of the safety report to the principal investigator, other institutions will be able to enroll patients in the study.

QOL is another interest during the treatment course of advanced HCC. Even if tumors respond to systemic chemotherapy leading to curative conversion surgery and prolonged survival, this may not always be linked to maintaining a high QOL. In contrast, noncurative surgery for symptomatic HCC after a response to chemotherapy may lead to a better QOL than systemic chemotherapy alone. Hence, a QOL assessment will be performed during the study period in the current study.

The atezo + bev regimen is expected to have a high response rate and conversion rate and to prolong the PFS of patients with advanced HCC. We plan to conduct a phase II trial to investigate the efficacy of conversion therapy with atezo + bev in patients with initially unresectable HCC. The results of the current study will help to establish treatment options for advanced HCC patients.

## Data Availability

Not applicable.

## List of abbreviations

HCC: hepatocellular carcinoma
atezo + bev: atezolizumab and bevacizumab
RECIST: Response Evaluation Criteria in Solid Tumors
PFS: progression-free survival
ICG: indocyanine green
TACE: transarterial chemoembolization
OS: overall survival
ORR: objective response rate
ICG-R15: indocyanine green retention rate at 15 min
QOL: quality of life
PS: performance status
CT: computed tomography
MRI: magnetic resonance imaging
PD: progression of the disease
CR: complete response
PR: partial response
CTCAE: Common Terminology Criteria for Adverse Events
BCLC: Barcelona Clinic Liver Cancer
